# Supplementary material for: Standardized Complex Gut Microbiomes Influence Fetal Growth, Food Intake, and Adult Body Weight in Outbred Mice
Source: Microorganisms. 2023 Feb 15;11(2):484. doi: 10.3390/microorganisms11020484 (PMC9961083; doi:10.3390/microorganisms11020484)
Supplement: Supplementary file 1 [file microorganisms-11-00484-s001.zip › Supplemental figures.pdf]

Supplemental figures

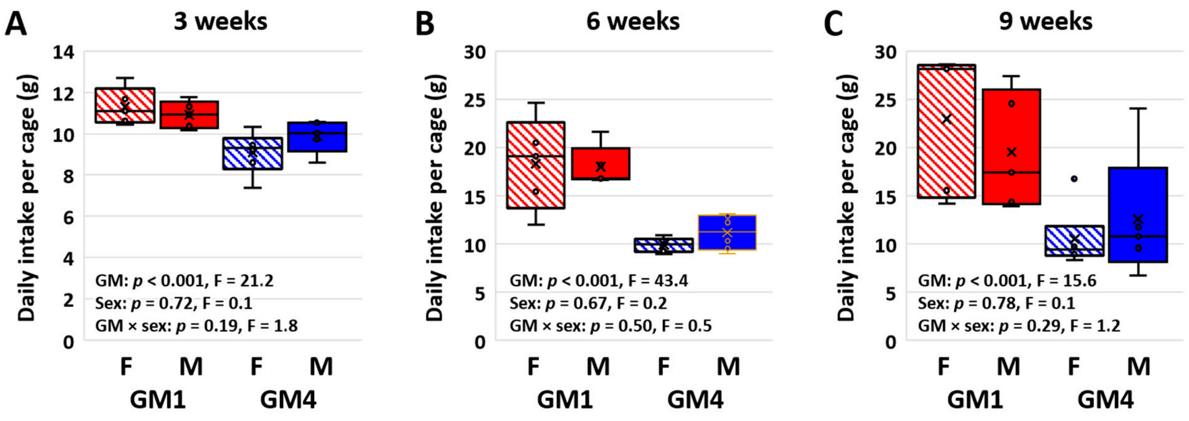

Figure S1. Box plots showing the total daily intake in cages housing pairs of female (F) or male (M) mice colonized with GM1 or GM4 at three (**A**), six (**B**) and nine (**C**) weeks of age (n = 5-6 cages/sex/GM). Statistical analysis performed using two-way ANOVA within each time-point.

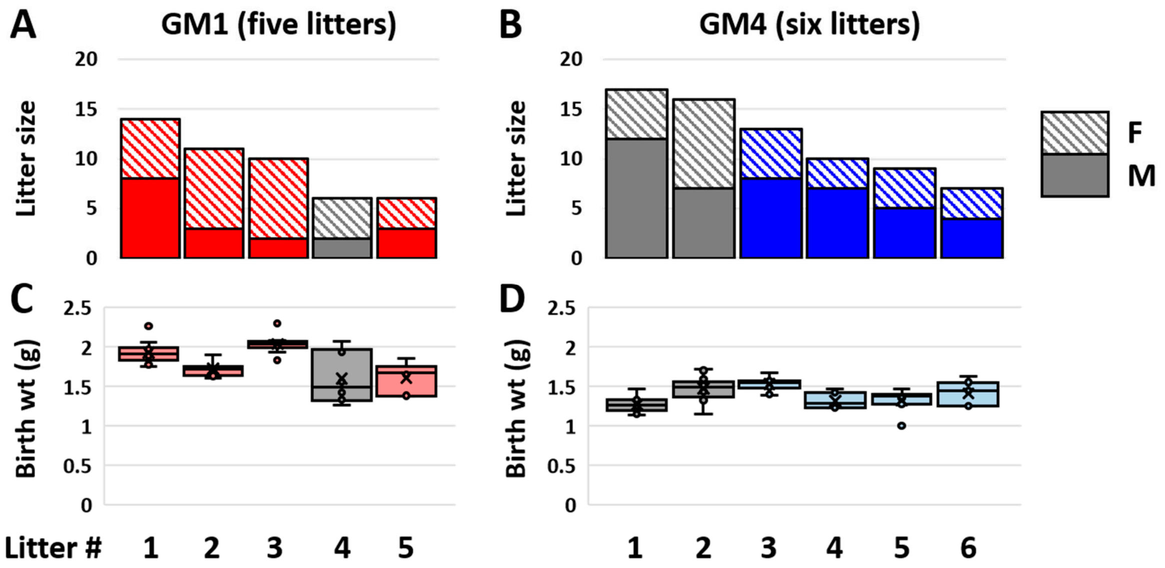

Figure S2. Bar plots showing the distribution of litter sizes (**A**) and sexes (**B**) in five or six litters, born to dams colonized by GM1 or GM4; and box plot showing birth weights of female (F) and male (M) mouse pups born to GM1-colonized (**C**) or GM4-colonized dams (**D**). Greyed out bars indicate litters removed from the analysis to control for litter size.

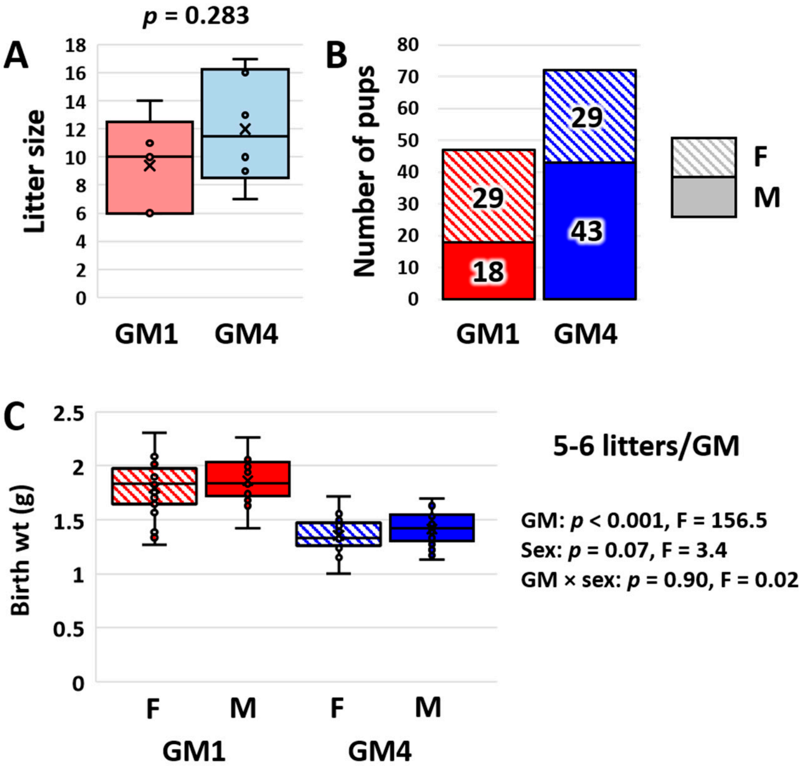

Figure S3. Box and bar plots showing the distribution of litter sizes (**A**) and sexes (**B**) in five or six litters, born to dams colonized by GM1 or GM4; and box plot showing birth weights of female (F) and male (M) mouse pups born to GM1- or GM4-colonized dams (**C**). Results of two-way ANOVA at right.

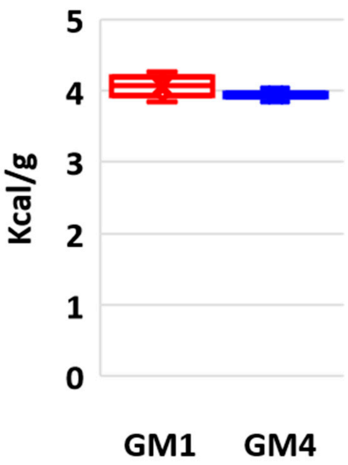

Figure S4. Energy content of freshly evacuated feces from adult female mice colonized with GM1 or GM4 (n = 3/GM).
